# Overcoming Challenges in Learning Prerequisites for Adaptive Functioning: Tele-Rehabilitation for Young Girls with Rett Syndrome

**DOI:** 10.3390/jpm15060250

**Published:** 2025-06-14

**Authors:** Rosa Angela Fabio, Samantha Giannatiempo, Michela Perina

**Affiliations:** 1 Department of Biomedical, Dental and Morphological and Functional Imaging Sciences, University of Messina, 98123 Messina, Italy; 2 Airett Centre for Research and Innovation, 37100 Verona, Italy; samantha.giannatiempo@centrotice.it (S.G.); michela.perina@airett.it (M.P.)

**Keywords:** Rett Syndrome, telerehabilitation, adaptive functioning, learning prerequisites, eye contact, engagement, digital health, neurodevelopmental disorders, quantitative assessment, personalized medicine

## Abstract

**Background/Objectives**: Rett Syndrome (RTT) is a rare neurodevelopmental disorder that affects girls and is characterized by severe motor and cognitive impairments, the loss of purposeful hand use, and communication difficulties. Children with RTT, especially those aged 5 to 9 years, often struggle to develop the foundational skills necessary for adaptive functioning, such as eye contact, object tracking, functional gestures, turn-taking, and basic communication. These abilities are essential for cognitive, social, and motor development and contribute to greater autonomy in daily life. This study aimed to explore the feasibility of a structured telerehabilitation program and to provide preliminary observations of its potential utility for young girls with RTT, addressing the presumed challenge of engaging this population in video-based interactive training. **Methods:** The intervention consisted of 30 remotely delivered sessions (each lasting 90 min), with assessments at baseline (A), after 5 weeks (B1), and after 10 weeks (B2). Quantitative outcome measures focused on changes in eye contact, object tracking, functional gestures, social engagement, and responsiveness to visual stimulus. **Results:** The findings indicate that the program was feasible and well-tolerated. Improvements were observed across all measured domains, and participants showed high levels of engagement and participation throughout the intervention. While these results are preliminary, they suggest that interactive digital formats may be promising for supporting foundational learning processes in children with RTT. **Conclusions:** This study provides initial evidence that telerehabilitation is a feasible approach for engaging young girls with RTT and supporting adaptive skill development. These findings may inform future research and the design of controlled studies to evaluate the efficacy of technology-assisted interventions in this population.

## 1. Introduction

Rett Syndrome (RTT) is a severe neurodevelopmental disorder affecting approximately 1 in 10,000 females, and is primarily caused by mutations in the MECP2 gene [1,2]. RTT manifests as a combination of cognitive, behavioral, and motor impairments. The progression of Rett syndrome (RTT) is generally categorized into four distinct stages. The initial phase, known as early onset, emerges between 6 and 18 months of age, presenting subtle signs that can easily go unnoticed. During this period, affected children may exhibit a decline in interest in toys, decreased eye contact, and delays in achieving motor milestones such as sitting and crawling.

The second phase, referred to as rapid regression, typically occurs between the ages of one and four years. Throughout this stage, children progressively lose abilities they had previously developed. This phase is marked by the appearance of core RTT characteristics, including repetitive hand movements, breathing irregularities (breath-holding, hyperventilation), ataxia, bruxism, dysautonomia, dysautonomia, epilepsy [3], unexplained bouts of crying, and a noticeable reduction in social engagement [4,5]. RTT is also characterized by other features such as increasing difficulties in motor abilities (dyspraxia), and cognitive decline.

The third stage, known as the plateau phase, extends until approximately ten years of age. While movement difficulties persist, there may be some improvements in behavior. Some children may show slight improvements in their ability to use their hands and communicate.

The final stage, termed late motor deterioration, arises after the first decade of life and is characterized by worsening muscle weakness, increasing mobility challenges, joint complications, and the development of scoliosis.

As RTT is a progressive disorder, patients require continuous, individualized rehabilitation throughout their lives. Research supports the effectiveness of high-frequency, low-intensity rehabilitation, which leads to improvements in various domains, including motor and cognitive functions [6]. Early, personalized interventions are crucial in helping patients reach their full potential, with family and caregiver involvement being key to maximizing therapeutic outcomes [7].

A critical component of the assessment and rehabilitation of RTT patients is the evaluation of core neuropsychological skills and basic behaviors [8]. The early detection of neuropsychological deficits is essential, as it allows clinicians to identify atypical developmental trajectories and initiate timely evidence-based interventions.

These foundational prerequisite abilities are key to identifying early signs of atypical development and tracking the progression of symptoms. The crucial competencies assessed include spontaneous and prompted eye contact, the ability to focus on and track objects and faces, functional gestures, cooperation with requests, the ability to sit long enough to complete a task, object permanence, turn-taking, and the communication of basic needs [9,10]. These skills are essential for typical cognitive and social development and can represent early markers of neurodevelopmental delay, enabling the early identification of delays and allowing for timely interventions [11,12].

Moreover, the systematic evaluation of these competencies plays a vital role in tailoring individualized rehabilitation strategies. By mapping a patient’s specific profile of strengths and weaknesses, clinicians can design targeted interventions that promote neuroplasticity and maximize the improvement of residual skills.

Given the challenges associated with attention and interaction with digital stimuli in RTT, concerns about the feasibility of engaging patients in remote interventions have arisen. However, emerging evidence suggests that tele-rehabilitation (TR) can be an effective method for improving cognitive, emotional, motor, and adaptive abilities in children with RTT [9,13,14]. By overcoming barriers to rehabilitation, such as limited physical access to specialized care and challenges in patient engagement, TR offers a promising alternative.

TR utilizes digital tools such as video conferencing, wearable sensors, 3D cameras, eye-tracking systems, and virtual reality to provide personalized and accessible rehabilitation remotely. Studies have demonstrated that TR can improve adaptive skills and reduce caregiver burden by facilitating self-regulation and continuous feedback [15,16].

The use of digital equipment such as avatars, games and virtual reality in RTT is recognized as a useful strategy to engage patients’ attention and executive functions and increase their motivation [17]. To assess the engagement and motivation of the participants a specific measure called “Motivation Index” is used. Through an analysis of the behavior of the participant, a scoring detects his/her grade of engagement [18,19].

Recent advancements in RTT rehabilitation emphasize the development of highly individualized intervention strategies tailored to the unique clinical profile of each patient. This includes a detailed characterization across genomic, biochemical, and behavioral dimensions to address the substantial inter-individual variability observed in Rett Syndrome [20,21,22]. Within this framework, personalized approaches are gaining traction in the treatment of neurodevelopmental disorders, aiming to support cognitive, social, and motor development through the enhancement of core neuropsychological abilities [23].

The present study aims to investigate the feasibility of delivering a structured tele-rehabilitation (TR) program to young girls with RTT and to provide preliminary data on changes in adaptive learning prerequisites over time. Specifically, the study focuses on assessing the development of key skills such as eye contact, object tracking, functional gestures, social engagement, and responsiveness to visual stimuli. By examining participants’ engagement and behavioral responses across the intervention period, this research seeks to contribute to the growing body of literature on the potential utility of tele-rehabilitation in supporting early neuropsychological development in RTT.

## 2. Materials and Methods

### 2.1. Participants

The Italian Rett Association (AIRETT) recruited 11 young girls with RTT, ranging from age 4 to 11 years old (Median: 7; IQR: 4.0–9.5). According to the criteria for classic RTT established by Hagberg et al. [24], participants were classified as clinical stage III. A general assessment was carried out by a psychologist using the Rett Assessment Rating Scale (RARS) [25] and the Vineland Adaptive Behavior Scale (VABS) [26]. Table 1 shows the characteristics of the participants.

The inclusion criteria were children aged 3–9, those with the ability to sit on a chair with support to ensure they could handle the TR sessions, and those without drug-resistant epilepsy. The exclusion criteria were related to the presence of CDKL5 or FOXG1 mutations. All procedures performed in studies were conducted in accordance with the ethical standards and approved by the Institutional Ethics Committee of Clinical and Experimental Medicine Department, University of Messina (protocol code 0135087 of 25 October 2023); the work was carried out in accordance with the Declaration of Helsinki as revised in 2000.

### 2.2. Study Design

**Figure 1:**
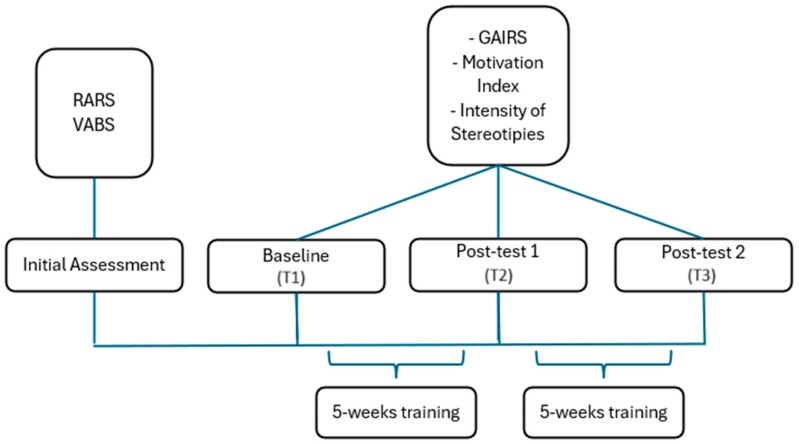
Study design.

This study employed a pre-test, post-test 1, and post-test 2 design, as shown in Figure 1. Participants underwent evaluation and treatment using a telerehabilitation (TR) program designed specifically for individuals with Rett Syndrome.

**Digital Health Intervention Description**

The telerehabilitation (TR) system was delivered via Cisco Webex, a secure video conferencing platform designed for professional remote collaboration and compliant with the General Data Protection Regulation (GDPR). Cisco Webex was selected for its robust privacy features, high reliability, and ability to maintain stable audio–video communication even under low-bandwidth. The platform enabled real-time, synchronous interaction between the therapist and participant, supporting the delivery of individualized cognitive tasks and therapist-guided exercises. All items required by the iCHECK-DH reporting standard are addressed in Appendix B [27].

To ensure accessibility, each participant joined the sessions using a standard laptop (Alienware model). Laptops were provided by the research team to guarantee equal access to the intervention across all households. The devices were pre-configured with the necessary software and tested before the start of the program.

Caregivers received initial training on how to use the Webex platform through an online demonstration and a printed manual with visual step-by-step instructions (e.g., how to connect, adjust settings, and troubleshoot common issues). Throughout the program, technical support was available on request to address any connectivity or usability problems.

The TR protocol was designed to be flexible and adaptable. If a participant was unable to attend a scheduled session due to health or technical issues, the session was rescheduled within the same week to ensure full participation. Thanks to this flexible format, all participants completed the 10-week intervention as planned, except for one girl who was hospitalized during the program and subsequently excluded from the final sample.

**Implementation Context**

Participants were recruited through the Italian Rett Syndrome Association (AIRETT), and all interventions were conducted in the participants’ homes. Families were provided with a detailed user manual and a 2 h training session to ensure the proper use of the digital platform and equipment. Technical support was available via phone and email throughout the intervention.

**Participants and Baseline Assessment**

After signing the informed consent to participate in the study, participants underwent an initial in-person or remote assessment conducted by certified therapists trained in Rett Syndrome. Standardized tools including the RARS (Rett Assessment Rating Scale) and the VABS (Vineland Adaptive Behavior Scales) were used for initial profiling.

Following the initial assessment, caregivers received training on how to use the TR platform, including device setup, session scheduling, and how to manage minor technical difficulties. The baseline assessment was then conducted remotely using the GAIRS checklist for global functioning, a rating scale for the intensity of stereotypies, and the Motivation Index. The same assessments were repeated after 5 weeks of training (post-test 1) and again at the end of the intervention, after 10 weeks (post-test 2). The assessments were then compared to evaluate the effects of the intervention.

**Intervention Delivery**

Participants engaged in a 10-week cognitive TR program, meeting remotely with a therapist three times per week for 1 h sessions. The therapist recorded performance and adherence data during each session using a digital tracking sheet built into the platform.

The cognitive training component included discrimination tasks adapted to each participant’s developmental level, based on the GAIRS Checklist results. The task difficulty progressed following a criterion of three correct responses across three sessions.

**Adaptability and Flexibility**

To accommodate potential health-related interruptions, such as hospitalizations or acute symptoms common in RTT, the intervention allowed for session rescheduling. One participant was excluded from the final sample due to hospitalization during the study. All others completed the program without major interruptions. No significant technical issues were reported during the TR sessions, and all caregivers reported the high usability of the platform.

**Sustainability and scalability**

The TR program was designed to be scalable across different regions and adaptable to individual needs. Since the intervention relied on commonly available devices (e.g., standard laptops) and a commercially available video conferencing platform (Cisco Webex), it can be feasibly extended to other families, care centers, or clinical contexts without requiring specialized infrastructure.

Furthermore, the training material for caregivers and the modular structure of the cognitive tasks allows for their replication and potential integration into broader rehabilitation programs. From a sustainability perspective, the low-cost nature of remote delivery, combined with the limited need for in-person resources, suggests that the intervention could be maintained over time, particularly if supported by local health services, patient associations (e.g., AIRETT), or telehealth funding schemes.

**Privacy and Data Security**

All digital interactions complied with Italian data protection laws and GDPR. Participant data were stored on encrypted servers and access was restricted to the research team. Caregivers were informed about data handling procedures and signed specific consent forms regarding digital data use.

### 2.3. Assessment and Measures

The initial assessment gathered information about the characteristics of the participants through RARS [26] and VABS [27] scales.

RARS is a standardized tool designed to evaluate patients with RTT to identify the severity of the disease. It is divided into 7 areas, namely cognitive, sensorial, motor, emotional, autonomy, typical characteristics and behavior. All 31 items represent the RTT profile of the patient. Every item can be scored from 1 to 4, where 1 means “within normal limits” and 4 means “strong abnormality”, and intermediate ratings are possible. The summing of the scores of all 31 items allows the evaluator, which can be a therapist or a caregiver, to identify the level of severity of RTT. This tool can identify a Mild severity (Score 0–55), Moderate severity (56–81) or Severe (>81). This instrument has been assessed as statistically valid and reliable. Specifically, normal distribution analyses of the scores were conducted, showing that the mean of the scale was close to both the median and the mode. The skewness and kurtosis values, calculated for the total score distribution, were 0.110 and 0.352, respectively, confirming the normality of the distribution. The internal reliability, measured using Cronbach’s alpha, was 0.912, while the internal consistency of the subscales was high, ranging from 0.811 to 0.934.

VABS is divided into four domains: communication, daily living, socialization, and motor skills. The interviewer asks general questions about the patient’s functioning in each domain and converts the responses into a score on each item like this: 2 = always present, 1 = sometimes present, 0 = seldom or never present. A typical interview lasts approximately one hour. A total score is obtained by summing the individual ratings for each scale. The reliability of the VABS was established as follows: the split-half reliability ranged from 0.73 to 0.93 for the communication domain, 0.83 to 0.92 for daily living skills, 0.78 to 0.94 for socialization, 0.70 to 0.95 for motor skills, 0.84 to 0.98 for the adaptive behavior composite, and 0.77 to 0.88 for maladaptive behavior. The interrater reliability coefficients for the survey and expanded forms ranged from 0.62 to 0.75. The standard error of measurement varied from 3.4 to 8.2 across the four domains and from 2.2 to 4.9 for the Adaptive Behavior Composite on the survey form.

At the baseline, at post-test 1 and at post-test 2, participants were assessed by a global evaluation using the GAIRS (Global Assessment and Intervention Rating Scale) [10], a checklist designed for RTT that merges the items of different scales for neurodevelopmental disorders and multi-disability [25,26,27,28,29,30,31,32,33]. It presents a global overview of areas of function to assess the overall abilities of the subject. It is made of 10 areas, which are basic pre-requisites, neuropsychological abilities, basic cognitive concepts, advanced cognitive concepts, communication abilities, emotional–affective abilities, hand motor skills, graphomotor skills, global motor abilities, and the level of autonomy in daily life.

In this study, the focus was on the basic pre-requisite abilities for learning, which is the first area of the GAIRS Checklist. Items of this area are listed in the table below (Table 2).

In the same context, the Motivation Index was assessed along with the number of aids required to complete a task.

**Figure 2:**
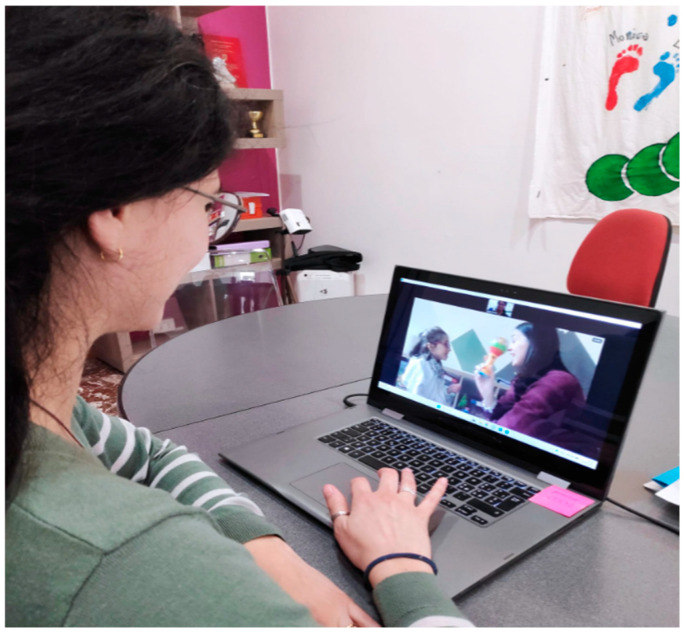
Example of a TR cognitive session in which the caregiver asks the child to track an object.

**Figure 3:**
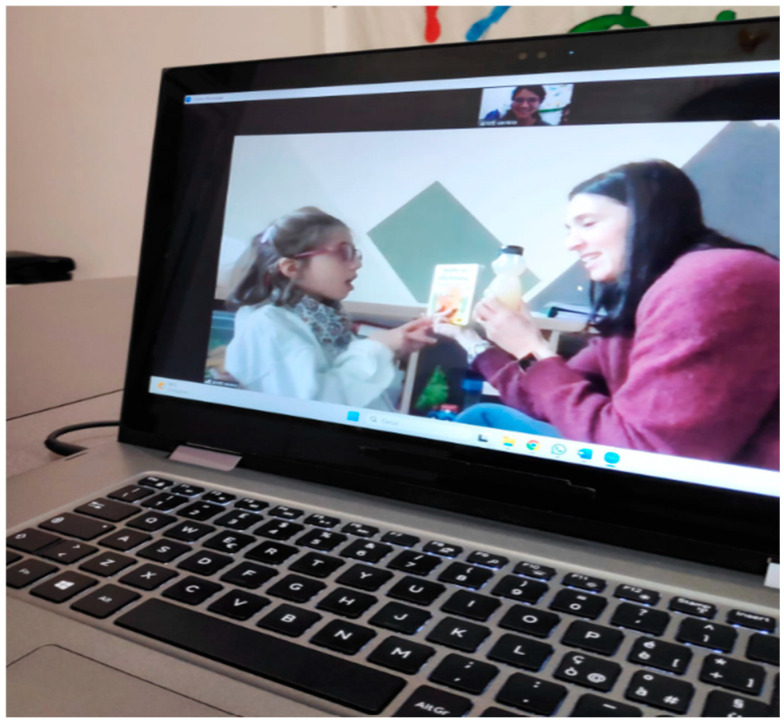
Example of a TR cognitive session in which the participant expresses a basic need using body language. The participant, seated in front of the therapist, indicates thirst by looking at the juice.

The Motivation Index comes from the taxonomy of Van der Maat [34], based on analyzing the behavior of people with profound intellectual disabilities with their caregivers. This taxonomy includes twelve primary categories of behavioral forms: gaze direction, facial expression, sounds, head posture, head movement, body posture, movements of the lower limbs, movements of the upper limbs, mouth movements, physiological reactions, aggression and conventional gestures. To create the Motivation Index, only five categories were considered, i.e., gaze direction, vocalizations, mouth movements, physiological responses (such as blushing or sweating), and hand gestures. The participant was recorded with a camera placed in front of her during the assessment and sessions (see Figure 2 and Figure 3). On a checklist, the presence of a behavior was marked as “1” and its absence as “0.” Two independent blind observers recorded the scores, and the MI was calculated as the total score across all behaviors. In this study, the agreement between the two observers was 96%.

The number of aids refers to the external supports and prompts provided by the caregiver to help the girls with Rett Syndrome maintain attention, manage stereotypies, and complete the task.

### 2.4. Procedure

All participants were recruited through the Italian Rett Syndrome Association (AIRETT). After signing the informed consent to participate in the study, they underwent an initial assessment, which included the RARS and VABS, administered by a trained professional. All professionals had certified training in RTT.

Following the initial assessment, training was provided for families or primary caregivers to ensure they could use the necessary TR equipment. The baseline assessment was then conducted, during which the global function of the participants was evaluated using the GAIRS checklist, the intensity of stereotypies, and the motivation index.

After the baseline assessment, the participant underwent 10 weeks of training using TR, meeting remotely with the therapist three times a week (see Figure 2 and Figure 3). The therapist collected data on the girls’ performance during each session, which lasted one hour. A post-test assessment (Post-test 1) was conducted after 5 weeks of training and new goals were established, followed by a second post-test (Post-test 2) at the end of the 10-week period.

Cognitive training involved cognitive discrimination tasks, tailored to the participant’s level, as assessed by the pre-requisites GAIRS Checklist. Progression to the next step followed a consistent criterion: three correct responses obtained in each of three treatment sessions.

To ensure full participation in the program, if a participant missed one of the three scheduled weekly sessions, the therapist rescheduled the session to a non-training day. This allowed all participants to complete the entire 10-week intervention as planned. One participant was hospitalized during the intervention period and was therefore excluded from the final sample. As individuals with Rett Syndrome may occasionally experience health-related issues, the program was designed to be flexible to accommodate such circumstances. No relevant technical issues were reported during the telerehabilitation sessions.

### 2.5. Statistical Analysis

The data for each GAIRS subscale were obtained following standardized instructions [35], and the mean score for each patient was calculated for the subscales, which range from 1 to 5 (as described in detail in Appendix A). Higher scores indicated that the patient had achieved mastery performance in that subscale.

Data analysis was conducted using IBM SPSS Statistics, Version 24 (IBM, Armonk, NY, USA). The assumption of normality was assessed using the Shapiro–Wilk test and a visual inspection of the data distribution. The results indicated that all variables were normally distributed across the three time points (T1, T2, T3), as none of the Shapiro–Wilk tests were significant (all *p* > 0.05; W ranges: 0.92–0.98). Given the assumption of normality, a 10 (prerequisite type) × 3 (time: baseline, post-test 1, post-test 2) within-subjects ANOVA was conducted to examine the effects of telerehabilitation over time and individual differences in performance. When significant effects were found, the effect size was reported. Effect sizes were computed and categorized based on eta squared (η^2^) [34]. For the paired-samples *t*-tests conducted to compare performance between time points, the effect sizes were calculated using Cohen’s d, with values interpreted according to conventional benchmarks (small: 0.2, medium: 0.5, large: 0.8). These effect sizes complemented the ANOVA results by providing a measure of the magnitude of change between specific time points.

## 3. Results

The descriptive statistics for each prerequisite at T1, T2, and T3 are reported in Table 3.

The within-subjects ANOVA revealed a significant main effect of time on all ten prerequisites (*p* < 0.05, with η^2^ ranging from 0.06 to 0.15), indicating that telerehabilitation had a positive impact on participants’ adaptive learning skills. Post hoc pairwise comparisons showed significant improvements from T1 to T2 (*p* < 0.01) and from T1 to T3 (*p* < 0.01) for most variables, while changes from T2 to T3 were generally smaller and mostly non-significant.

Paired-samples t-tests further explored these differences and provided estimates of the magnitude of change between time points (Table 3). Significant improvements with moderate to large effect sizes were observed from T1 to T2 and from T1 to T3 for several prerequisites, including spontaneous eye contact (T1 vs T2: *t*(10) = 2.21, *p* < 0.01, *d* = 0.56; T1 vs. T3: *t*(10) = 2.23, *p* < 0.01, *d* = 0.67), eye contact on request (T1 vs. T2: *t*(10) = 2.11, *p* < 0.05, *d* = 0.65; T1 vs. T3: *t*(10) = 2.24, *p* < 0.05, *d* = 0.68), object permanence (T1 vs. T2: *t*(10) = 3.11, *p* < 0.01, *d* = 0.78; T1 vs. T3: *t*(10) = 3.40, *p* < 0.01, *d* = 1.03), and turn-taking (T1 vs. T2: *t*(10) = 2.31, *p* < 0.05, *d* = 0.84; T1 vs. T3: *t*(10) = 2.81, *p* < 0.01, *d* = 0.85). Changes between T2 and T3 were generally smaller and not statistically significant, suggesting that most gains occurred in the first phase of the intervention.

**Figure 4:**
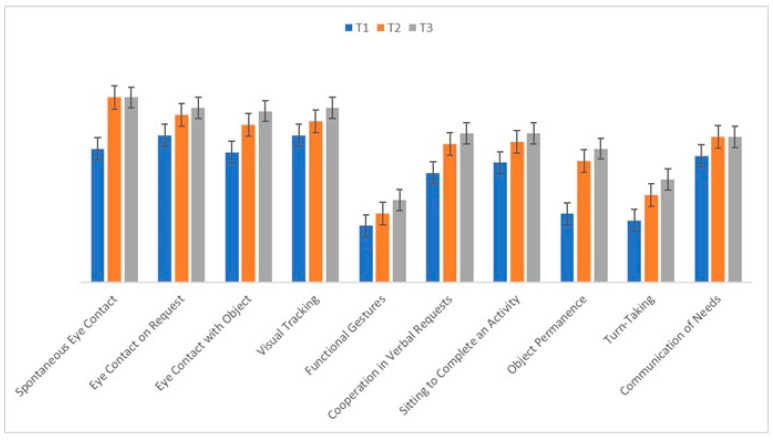
Increments in each area of the prerequisites. The bar chart illustrates the improvements observed across various prerequisite areas, with higher values indicating greater progress in the respective domain.

These results confirm the significant improvements detected by the ANOVA and emphasize that telerehabilitation effectively enhanced adaptive learning prerequisites over time, with the largest changes occurring early in the intervention period. Figure 4 illustrates these overall trends, while Table 3 details the pairwise comparisons with associated statistics.

### 3.1. Unpacking the Trend: Exploring Individual Differences

**Figure 5:**
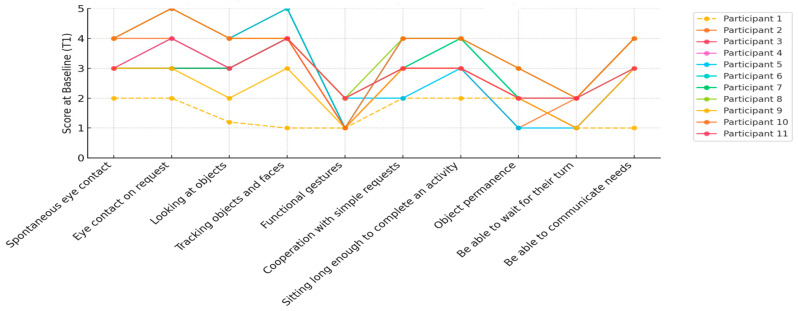
Individual participant trajectories in each area of the prerequisites at baseline (T1).

**Figure 6:**
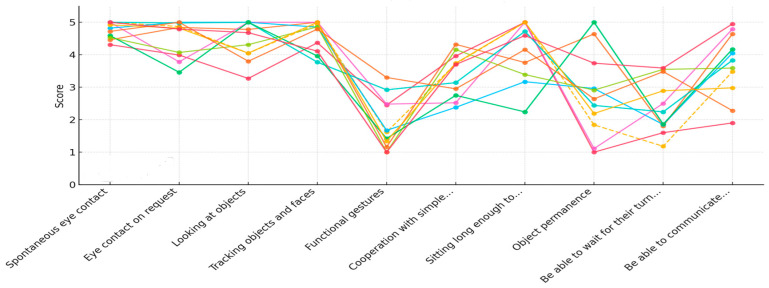
Individual participant trajectories in each area of the prerequisites at post-test 1 (T2); each color represents a different participant.

**Figure 7:**
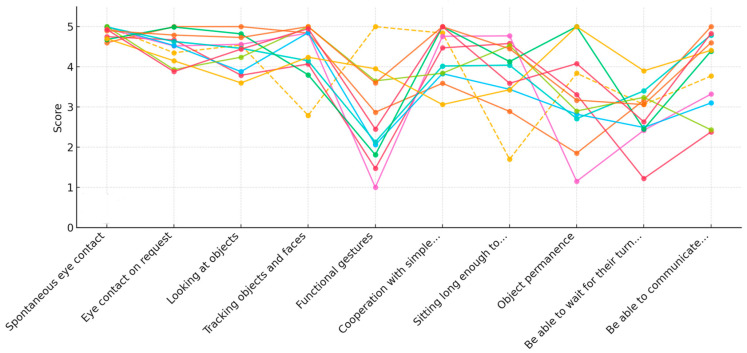
Individual participant trajectories in each area of the prerequisites at post-test 2 (T3), each color represents a different participant.

To better illustrate the individual variability across participants—particularly considering the known heterogeneity in RTT—we also present a graph of the individual participant trajectories across time for all prerequisites (see Figure 5, Figure 6 and Figure 7). This visualization highlights the consistency of the observed improvements and the range of responses to the intervention.

As illustrated by the dashed line corresponding to one participant, referred to here by the pseudonym Flavia, she exhibited typical development until 20 months of age, after which she experienced the characteristic regression associated with Rett Syndrome (RS), progressing through its four typical stages.

At the baseline assessment (Figure 5), her performance in the prerequisite skills domain indicated that, while she occasionally established spontaneous eye contact, she was unable to maintain eye contact upon request or track objects visually. Additionally, she demonstrated some functional gestures and intermittent cooperation with others. Her ability to remain seated for an extended period was also inconsistent, suggesting the need for further improvement in these areas.

After 10 weeks of intervention (Figure 7), notable progress was observed across multiple domains. Flavia demonstrated a consistent ability to establish spontaneous eye contact and, importantly, developed the capacity to respond with eye contact upon request. Her cooperative behavior and ability to remain seated for extended durations also showed significant improvement

### 3.2. Telerehabilitation and Motivation

Examining the role of telerehabilitation in enhancing motivation, *t*-tests showed significant results demonstrating increased motivation throughout the sessions (T1 vs. T2, *p* < 0.05; T2 vs. T3, *p* < 0.01), along with a reduction in the number of aids required to stay focused. Table 4 presents the means and standard deviations for the motivation index and the number of aids given across sessions 1, 5, and 10.

**Table 4:** Changes in motivation index and number of aids given across sessions.

Parameter	Session 1 (M ± SD)	Session 5 (M ± SD)	Session 10 (M ± SD)
Motivation index	4.31 ± 2.11	5.23 ± 0.91	6.00 ± 0.00
Number of Aids Given	32.16 ± 15	25.42 ± 13.9	18.1 ± 12.91

In addition to the quantitative results, qualitative observations indicated that the participants exhibited high levels of engagement throughout the intervention. Parents and caregivers reported increased willingness to participate in interactive tasks, reduced frustration, and improved social responsiveness. These findings suggest that, despite initial concerns about digital engagement, young girls with RTT can benefit substantially from structured telerehabilitation programs.

Overall, the results demonstrate that telerehabilitation can be a powerful tool for enhancing key learning prerequisites in young girls with RTT. The improvements observed across multiple adaptive domains highlight the potential of remote interventions in fostering cognitive, social, and motor development, paving the way for further research on personalized, technology-assisted rehabilitation approaches.

## 4. Discussion

This study explored the feasibility and preliminary efficacy of a structured telerehabilitation (TR) program in enhancing key adaptive and neuropsychological prerequisites in young girls with Rett Syndrome (RTT). Overall, our findings support the potential use of TR as a viable and engaging method to promote the foundational skills critical for cognitive, social, and motor development in RTT. Significant improvements were observed across multiple domains—including eye contact, turn-taking, and object permanence—highlighting the promise of remote, individualized interventions for this clinical population.

The observed improvements align with existing literature emphasizing the importance of early, personalized interventions in neurodevelopmental disorders [1,2,6]. The significant main effect of time across all assessed prerequisites suggests that TR was effective in supporting the acquisition and reinforcement of essential developmental skills. Particularly noteworthy were the gains in object permanence and turn-taking—skills closely tied to social interaction and executive function—underscoring the value of interactive digital tools in engaging RTT patients.

This study also highlighted the importance of personalized approaches in TR delivery. RTT is characterized by substantial inter-individual variability in clinical presentation, requiring flexible and individualized rehabilitation strategies [20,21,22]. Our intervention was tailored to each child’s unique profile, with adaptive adjustments made based on their baseline abilities and progress. The integration of tools such as avatars, gamified tasks, and video conferencing enabled a dynamic rehabilitation environment that supported sustained attention, engagement, and skill development.

These findings are consistent with a growing body of research advocating for the use of digital technologies in neurorehabilitation [13,14,15,16]. Digital tools not only increase the accessibility and continuity of care, particularly for rare disorders such as RTT, but also enable the implementation of interactive and motivating formats that are often difficult to reproduce in traditional in-person settings. The positive feedback from caregivers regarding children’s increased willingness to participate and reduced frustration further supports the acceptability and practical relevance of this approach.

However, several important limitations must be acknowledged. First and foremost, this study did not include a control group. As such, while we observed significant improvements over time, we cannot conclusively attribute these gains solely to the TR intervention. The natural progression of RTT, spontaneous fluctuations, or other concurrent factors (e.g., parental support, medication changes) could have influenced the outcomes. We explicitly acknowledge this limitation and recognize the need for future controlled studies to establish causal effects. Second, the sample size was small, limiting the generalizability of the findings and ability of the statistical power to detect more nuanced effects or interactions. Third, the inclusion and exclusion criteria may have further restricted the applicability of the results to the broader RTT population, as participants who could not engage with digital tools or sustain attention for a minimum duration were not included.

Despite these limitations, the findings provide preliminary but promising evidence supporting the feasibility and utility of personalized telerehabilitation in RTT. Importantly, this study contributes to the literature by demonstrating that even very young girls with RTT—who are often considered difficult to engage—can benefit from structured, remote programs that emphasize core developmental skills. These results support ongoing efforts to develop accessible, technology-assisted interventions tailored to the specific needs of individuals with neurodevelopmental disorders.

In conclusion, the results underscore the potential of telerehabilitation as an accessible, flexible, and effective approach to supporting early developmental skills in RTT. While further research—including randomized controlled trials with larger samples—is needed to establish efficacy, the present findings offer encouraging evidence that individualized TR programs can promote meaningful gains in the foundational abilities essential for long-term adaptive functioning. The integration of TR into clinical practice may represent a valuable component of personalized care strategies for RTT, contributing to more inclusive, scalable, and responsive models of neurodevelopmental rehabilitation.

## Figures and Tables

**Table 1:** Characteristics of participants.

Participants	Name	Clinical Stage	Age	MeCP2 Mutation	Level of Severity (RARS)	Functional Ability (VABS)
1	C.A.	III	4	T158M	58	84
2	A.C.	III	5	T158M	71	71
3	C.L.	III	3	P152R	69.5	109
4	C.M.	III	7	P322L	65.5	104
5	B.C.	III	5	R255X	71	75
6	S.A	III	4	P322L	75	108
7	G.L	IV	9	R255X	75.5	84
8	S.L	IV	9	T158M	70	78
9	B.A	III	9	P152R	75	71
10	P.V	III	8	R255X	65.5	69
11	L.M	III	9	P322L	58	136

**Table 2:** Items of the Pre-requisites Area of GAIRS Checklist and the respective assessment procedure *.

Ability	Assessment Procedure
Spontaneous Eye Contact	The participant sits in front of the therapist. The therapist talks to the participant who must spontaneously look at him/her for a few seconds.
Eye Contact on Request	The participant sits in front of the therapist. The therapist calls the participant or says, “Look at me!” in an enthusiastic way and the participant has to look at him/her for a few seconds.
Ability to Look at Objects	The participant sits in front of the therapist. The therapist shows an object (e.g., a ball) and says, “Look at the ball!” and the participant has to look at the ball for a few seconds.
Tracking Objects and Faces	The participant sits in front of the therapist. The therapist shows an object (e.g., a ball) or her/his face to the participant and says “follow the direction of the ball!” or “Follow me” while he/she moves the object in the space from “up” to “down” or from “right” to “left” and vice versa and the participant has to follow it. The participant can also follow the movement of the therapist in the space for a few seconds.
Functional Gestures	The participant sits at a table in front of the therapist. The therapist puts a motivating object on the table (e.g., a preferred object or something edible) and asks the participant to approach, point, and give it.
Cooperation with Simple Spoken Request	The participant sits in front of the therapist. The therapist asks the participant a simple spoken request like reply to her/his name or look for mother and tests if the participant understands this request.
Ability to Sit Long Enough to Complete a Task	The participant sits at a table in front of the therapist. The therapist asks the participant to stay until she/he has completed a simple task depending on the type of tasks (5 min, 2 min or 1 min).
Object Permanence	The participant sits at a table in front of the therapist. The therapist presents a preferred object or food item, then hides it under a cup or tissue, and asks the participant to find it, look at it, and take it (if the participant has the necessary motor skills).
Ability to Wait for Their Turn before Starting an Activity	The participant sits in front of the therapist, who prepares an activity and asks the participant to wait for a few seconds, depending on the time needed to set up the task (e.g., 10 s, which is the time it takes for the therapist to present two targets in a discrimination task).
Ability to Communicate Basic Needs	The participant sits at a table in front of the therapist and expresses a basic need using body language (e.g., looking at a water bottle to indicate thirst or touching their stomach to indicate hunger) or facial expressions (e.g., to communicate pain). The participant can do this either by looking alone or by both looking and touching.

* The detailed scoring method is in Appendix A.

**Table 3:** Means and standard deviations (SD) of each prerequisite, with statistical comparisons between phases.

Learning Prerequisites	T1 (SD)	T2 (SD)	T3 (SD)	T1 vs. T2	T1 vs. T3	T2 vs. T3
Spontaneous Eye Contact	3.55 (0.69)	4.91 (0.30)	4.91 (0.30)	t(10) = 2.21, *p* < 0.01, d = 0.56	t(10) = 2.23, *p* < 0.01, d = 2.56	t(10) = 0.01, *p* = 1, d = 0.00
Eye Contact on Request	3.91 (0.94)	4.45 (0.69)	4.64 (0.50)	t(10) = 2.11, *p* < 0.05, d = 0.65	t(10) = 2.24, *p* < 0.05, d = 0.97	t(10) = 0.18, *p* = 0.87, d = 0.32
Eye Contact with Object	3.45 (1.04)	4.18 (0.75)	4.55 (0.52)	t(10) = 1.98, *p* < 0.05, d = 0.51	t(10) = 2.53, *p* < 0.01, d = 1.34	t(10) = 0.82, *p* = 0.34, d = 0.57
Visual Tracking	3.91 (0.83)	4.27 (0.79)	4.64 (1.03)	t(10) = 1.12, *p* = 0.08, d = 0.44	t(10) = 2.34, *p* < 0.05, d = 0.78	t(10) = 1.08, *p* = 0.41, d = 0.40
Functional Gestures	1.50 (0.81)	1.82 (0.84)	2.18 (1.21)	t(10) = 0.88, *p* = 0.67, d = 0.39	t(10) = 1.99, *p* < 0.05, d = 0.66	t(10) = 1.23, *p* = 0.23, d = 0.35
Cooperation in Verbal Requests	2.91 (0.94)	3.68 (0.84)	3.96 (0.72)	t(10) = 2.23, *p* < 0.05, d = 0.66	t(10) = 2.40, *p* < 0.01, d = 0.71	t(10) = 0.76, *p* = 0.34, d = 0.36
Sitting to Complete an Activity	3.18 (0.99)	3.73 (1.08)	3.96 (0.88)	t(10) = 1.11, *p* = 0.23, d = 0.53	t(10) = 1.89, *p* < 0.05, d = 0.83	t(10) = 0.22, *p* = 0.76, d = 0.23
Object Permanence	1.82 (0.75)	3.23 (1.33)	3.55 (1.04)	t(10) = 3.11, *p* < 0.01, d = 0.78	t(10) = 3.40, *p* < 0.01, d = 0.91	t(10) = 0.31, *p* = 0.54, d = 0.27
Turn-Taking	1.64 (0.67)	2.32 (0.93)	2.73 (0.85)	t(10) = 2.31, *p* < 0.05, d = 0.84	t(10) = 2.81, *p* < 0.01, d = 0.71	t(10) = 0.42, *p* = 0.25, d = 0.46
Communication of Needs	3.36 (0.92)	3.86 (0.95)	3.86 (0.95)	t(10) = 0.72, *p* = 0.38, d = 0.53	t(10) = 0.76, *p* = 0.37, d = 0.53	t(10) = 0.0, *p* = 1, d = 0.00

## Data Availability

The datasets analyzed and generated during the study are provided.

## Abbreviations

The following abbreviations are used in this manuscript:

RTT	Rett Syndrome
RARS	Rett Assessment Rating Scale
VABS	Vineland Adaptive Behavior Scale
GAIRS	Global Assessment Rating Scale

## Appendix A

Scoring method for Prerequisite Skills.

**Table:**

Skill	1—Very Limited	2—Emerging	3—Developing	4—Consistent	5—Mastered
**Spontaneous Eye Contact**	The child is unable to establish spontaneous eye contact or does so only 1 time out of 10.	The child establishes spontaneous eye contact 2–3 times out of 10.	The child establishes spontaneous eye contact 4–6 times out of 10.	The child establishes spontaneous eye contact 7–8 times out of 10.	The child always establishes spontaneous eye contact.
**Eye Contact on Request**	The child does not look at the interlocutor/partner when requested or does so only 1 time out of 10.	The child looks at the interlocutor/partner 2–3 times out of 10 requests.	The child looks at the interlocutor/partner 4–6 times out of 10 requests.	The child looks at the interlocutor/partner 7–8 times out of 10 requests.	The child always looks at the interlocutor/partner.
**Looking at Objects**	The child does not look at an object or does so only 1 time out of 10 when requested.	The child looks at an object 2–3 times out of 10 requests.	The child looks at an object 4–6 times out of 10 requests.	The child looks at an object 7–8 times out of 10 requests.	The child always looks at an object when requested.
**Following Moving Objects or Faces**	The child does not follow a moving object or face or does so only 1 time out of 10 requests.	The child follows a moving object or face 2–3 times out of 10 requests.	The child follows a moving object or face 4–6 times out of 10 requests.	The child follows a moving object or face 7–8 times out of 10 requests.	The child always follows a moving object or face.
**Functional Gestures (Approaching, Pointing, Giving an Object)**	The child does not approach, point, or give an object, or does so only 1 time out of 10.	The child approaches, points, or gives an object 2–3 times out of 10 requests.	The child approaches, points, or gives an object 4–6 times out of 10 requests.	The child approaches, points, or gives an object 7–8 times out of 10 requests.	The child always approaches, points, or gives an object when requested.
**Cooperation with Simple Spoken Requests**	The child does not understand simple spoken requests or follows them only 1 time out of 10.	The child follows simple spoken requests 2–3 times out of 10.	The child follows simple spoken requests 4–6 times out of 10.	The child follows simple spoken requests 7–8 times out of 10.	The child always follows simple spoken requests.
**Sitting Long Enough to Complete a Task**	The child is unable to sit at the table long enough to complete a task or does so only 1 time out of 10.	The child sits at the table long enough to complete a task 2–3 times out of 10 requests.	The child sits at the table long enough to complete a task 4–6 times out of 10 requests.	The child sits at the table long enough to complete a task 7–8 times out of 10 requests.	The child always sits at the table long enough to complete a task.
**Object Permanence (Finding a Hidden Object)**	The child cannot find a hidden object or does so only 1 time out of 10.	The child finds a hidden object 2–3 times out of 10.	The child finds a hidden object 4–6 times out of 10.	The child finds a hidden object 7–8 times out of 10.	The child always finds the hidden object.
**Waiting for Turn Before Starting an Activity**	The child is not able to wait for their turn before starting an activity.	The child waits for their turn 2–3 times out of 10 requests.	The child waits for their turn 4–6 times out of 10 requests.	The child waits for their turn 7–8 times out of 10 requests.	The child always waits for their turn before starting an activity.
**Communicating Basic Needs**	The child does not communicate basic needs in an understandable way.	The child communicates one basic need in an understandable way.	The child communicates 2–3 basic needs in an understandable way.	The child communicates 3–4 basic needs in an understandable way.	The child communicates all basic needs in an understandable way.

The table above provides a structured assessment of prerequisite skills. Each skill is scored from 1 to 5, reflecting increasing levels of competence: 1—Very Limited: The child rarely or never demonstrates the skill (1 time out of 10 or not at all). 2—Emerging: The child shows the skill inconsistently (2–3 times out of 10). 3—Developing: The child demonstrates the skill with moderate consistency (4–6 times out of 10). 4—Consistent: The child reliably performs the skill (7–8 times out of 10). 5—Mastered: The child always demonstrates the skill when required.

## Appendix B

iCHECK-DH Checklist.

**Table:**

Item	Description	Reported in Manuscript
1. Title and Abstract	The title and abstract should indicate that the study involves a digital health intervention.	Yes—the term “telerehabilitation (TR)” is used.
2. Background and Rationale	Provide context and justification for using a digital health approach.	Yes—TR is applied for patients with Rett Syndrome to increase accessibility.
3. Implementation Context	Describe the setting (e.g., home, clinic) and local context.	Yes—intervention took place at home with recruitment via AIRETT.
4. Implementation Objectives	Clearly define what the digital intervention aimed to achieve.	Yes—cognitive training, reduction in stereotypies, increased motivation.
5. Digital Health Intervention Description	Describe the platform, content, delivery method, and any tailoring.	Yes—cognitive tasks via remote therapist-led sessions using a secure platform.
6. Technology Platform	Name and features of the digital tool used (e.g., videoconferencing software).	Yes—Cisco Webex platform is used.
7. User Training and Support	Describe training for users and availability of support.	Yes—caregivers received manuals and live training; ongoing support was provided.
8. Access and Infrastructure	Specify how participants accessed the intervention (devices, connectivity).	Yes—all participants accessed the intervention through home internet access; Alienware laptops were provided by the research team.
9. Privacy and Security	Describe data protection strategies (encryption, GDPR compliance).	Yes—compliance with GDPR and use of encrypted storage mentioned.
10. Usability and Acceptability	Include data or observations on ease of use and user satisfaction.	Yes—caregivers reported high usability; no technical issues observed.
11. Adaptability	Explain how the intervention handled unexpected events (e.g., illness).	Yes—rescheduling sessions was allowed; one participant was excluded due to hospitalization.
12. Monitoring and Fidelity	Report how usage and adherence were monitored.	Yes—therapist recorded session data via digital logs.
13. Implementation Barriers and Facilitators	Describe what helped or hindered implementation.	Partially—no major issues were reported; high engagement noted.
14. Sustainability and Scalability	Describe if and how the intervention could be scaled or continued.	Yes—Described in Methods. Uses common devices and scalable procedures; training materials support sustainability.
15. Ethical Approval	Indicate that the study received appropriate ethics review.	Yes—Approval from University of Messina (protocol code 0135087 of 25 October 2023)
16. Funding and Conflicts of Interest	State sources of funding and potential conflicts.	Yes—No external fundings were received.
